# Austin District Medical Society

**Published:** 1887-11

**Authors:** 


### AUSTIN DISTRICT MEDICAL SOCIETY.

The following circular has been issued:

AUSTIN, TEXAS, NOV. 8th, 1887.

DEAR DOCTOR.—On the eighth of September last about twenty-five physicians of Travis and adjacent counties met in the city of Austin, and organized the "Austin District Medical Society," with the view to the futher development and promotion of medical science, and the establishing of a closer professional brotherhood.

The next meeting is fixed at Austin, for December 8, 1887.

The papers to be read for discussion on said occasion embrace the following subjects:

"Gastrotomy," a case, by Dr. J. A. Black, Round Rock.

"Puerperal Septicæmia," by Dr. L. D. Hill, Webberville.

"Typhoid Fever," by Dr. W. A. Morris, Austin.

"Errors of Refraction," by Dr. T. J. Tyner, Austin.

We earnestly and cordially invite you to meet with us on this occasion and participate in the discussions, which will be a most interesting feature; and, if you are not already a member to identify yourself at once with the Society.

Judging from the success of the initial meeting, we feel safe in saying that a right royal time will be had and much practical and useful work will be accomplished.

Fraternally yours,

THOS. D. WOOTEN, M.D.
T. J. BENNETT, M. D. Sec'y. President.

In addition to the above it is expected that Dr. F. T. Paine will be present, and read a peper on, or make some demonstrations in electro-therapeutics; and Dr. Menger, of San Antonio, has forwarded to Dr. Daniel, who will exhibit them on this occasion, a collection of micro-photographs of various subjects, — including blood corpuscles of various annimals.—Amongst the latter is one of the blood of a cow, suffering with fever, in the center of the corpuscles of which can be seen a micrococcus, said to be identical with that of dengue, first discovered by Dr. McLaughlin. Perhaps Dr. Menger may be present; if so, he will exhibit these micro-photographs, and explain them. Let all attend who can.

